# Infected Dentigerous Cyst and its Conservative Management: A Report of Two Cases

**DOI:** 10.5005/jp-journals-10005-1578

**Published:** 2019

**Authors:** Amol S Patil, Prasad N Jathar, Amey M Panse, Samhita R Bahutule, Raju U Patil, Madhuri Patil

**Affiliations:** 1,6 Department of Pediatric and Preventive Dentistry, MA Rangoonwala College of Dental Science and Research Centre, Pune, Maharashtra, India; 2–5 Department of Pediatric and Preventive Dentistry, Sinhgad Dental College and Hospital, Pune, Maharashtra, India

**Keywords:** Decompression, Infected dentigerous cyst, Mixed dentition, Radicular cyst, Stent

## Abstract

**Aim:**

To check the efficacy of decompression technique in children with dentigerous cysts.

**Background:**

Most commonly occurring odontogenic cysts in the oral cavity are radicular cysts and dentigerous cysts.^1^ According to Kramer, a cyst is defined as a pathological cavity having fluid, semi-fluid, or gaseous contents, which is not created by the accumulation of pus. A cyst which is lined by epithelium is known as a true cyst and that which is not lined by epithelium known as a pseudocyst.^2^ The dictionary meaning of dentigerous is “structures resembling teeth”.^3^ A dentigerous cyst is found enveloping the crown of an unerupted, embedded, or submerged tooth by the expansion of its follicle till the neck of the tooth.^1^ It is not unusual for a dentigerous cyst to mimic a radicular cyst, especially when the cyst is associated with a pulpectomized or carious primary tooth and its unerupted permanent successor. This article presents two cases of infected dentigerous cysts. The first case was of a female patient associated with tooth 45; and another case was of a male patient associated with tooth 35. The infected dentigerous cyst in both the cases was treated with the most conservative option available, i.e., decompression technique.

**Case description:**

In this article, two cases of infected dentigerous cysts are discussed, in which one case deals with the female patient associated with tooth 45 and the other case deals with the male patient associated with tooth 35. The infected dentigerous cysts in both the cases were treated conservatively, i.e., with the decompression technique.

**Conclusion:**

The present case report states that the decompression technique may be the most conservative method available for managing dentigerous cysts in children.

**How to cite this article:**

Patil AS, Jathar PN, *et al.* Infected Dentigerous Cyst and its Conservative Management: A Report of Two Cases. Int J Clin Pediatr Dent 2019;12(1):68–72.

## INTRODUCTION

In 1908, “Paget” coined the term “Dentigerous cyst”.^3^ A male preponderance is seen with a male to female ratio of around 1.84:1.^2^ Among all the true cysts of the jaws, dentigerous cyst accounts for around 24%,^4^ which is commonly seen in 2nd–3rd decades of life.^1^ In an Israeli study, the incidence of dentigerous cyst was around 45% in pediatric patients.^2^ However, the frequency of radicular cyst occurrence in primary dentition is rare accounting for around 0.5–3.3%.^5^ The purpose of this paper is to present the management of an infected odontogenic cyst (dentigerous cyst) and its conservative modality.

## CASE DESCRIPTION

In the present case, the diseased primary molar was extracted to gain a conservative access to the cystic site and decompression was chosen as the modality of choice since the preservation of the permanent successor was desirable.

## CASE 1

**Figs 1A and B:**
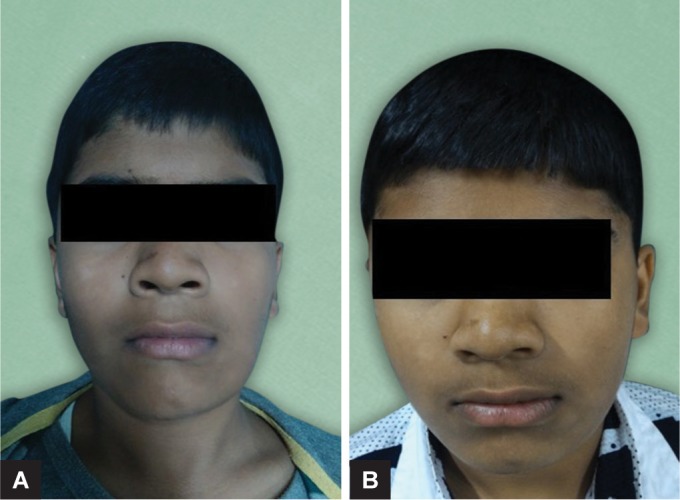
Extraoral photographs of case 1: (A) Preoperative photograph; (B) Postoperative photograph

**Figs 2A to C:**
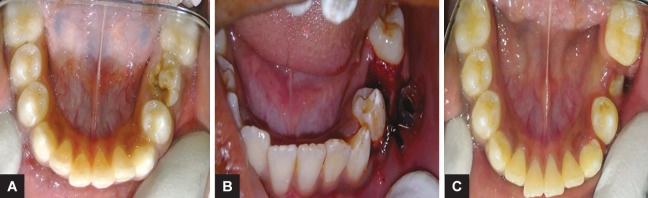
Intraoral photographs of case 1: (A) Preoperative photograph; (B) Intraoperative photograph; (C) Postoperative photograph

A 12-year-old male patient was reported to the Outdoor Patient Department of Pediatric and Preventive Dentistry, Pune, Maharashtra, with a chief complaint of pain and swelling in the lower left back region of the jaw since 15 days. Medical history was non-contributory. Extra-orally facial asymmetry was noted on the left side of the jaw with swelling extending anterio-posteriorly from the angle of the mouth to the lower border of the mandible (Fig. 1). The swelling had a smooth surface and the color of the swelling over the skin was normal. Palpatory findings revealed enlarged left submandibular lymph nodes tender on palpation. Intraoral examination revealed the expansion of the buccal cortical plate extending from the distal side of the left mandibular first premolar to the distal side of the first molar, measuring approximately 3 × 3 cm. The overlying mucosa was hard, red, and tender on palpation. It was smooth and no ulceration was observed. Also, the involved primary tooth was carious (Fig. 2). The panoramic view revealed unilocular radiolucency, which was well-defined and corticated extending from the distal surface of the mandibular left first premolar root to the distal surface of the mandibular first molar also involving the impacted second molar. Based on the above findings, a provisional diagnosis of radicular cyst was given.

**Figs 3A to C:**
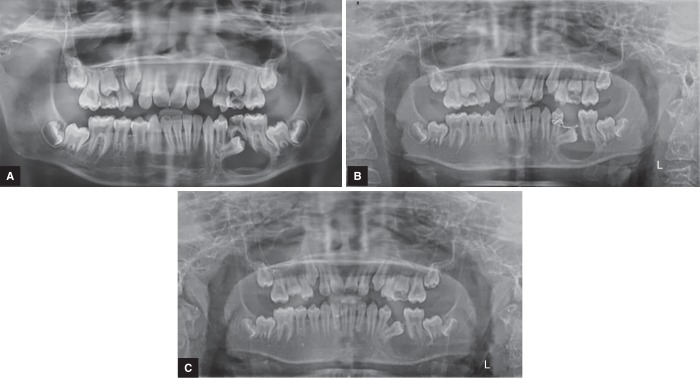
Panoramic radiographs: (A): Preoperative OPG; (B) Intraoperative OPG; (C) Postoperative OPG

To confirm the provisional diagnosis, fine needle aspiration cytology (FNAC) was planned. The procedure to be done was explained to the parents and the patient and their consent was obtained. The FNAC sample was sent to the Department of Oral Pathology and extraction of 75 was planned. Extraction of the involved primary tooth was carried out under local anesthesia (LIGNOX 2%), followed by aspiration of the cystic fluid and further irrigation with povidone iodine 5% (Indiamart). The socket was curated and the sample along with the extracted tooth was sent for confirmatory diagnosis. To prevent the loss of the impacted permanent premolar, the decompression technique was chosen as the treatment of choice. The cystic cavity was opened through the alveolar socket of 75, which helped to relieve the intra-cystic pressure of the cystic cavity further decreasing its size.^6^ A plastic stent made up of a needle cap was used to keep the cystic cavity in contact with the oral cavity and to open it for irrigation. The stent was stabilized using a ligature wire (26 gauge) and the patient was recalled after one week. A slight reduction in swelling and tenderness was noticed after 1 week. After 3 weeks recall, clinical and radiographic examination (orthopantomograph (OPG)), a gradual reduction in swelling and tenderness was observed (Fig. 1). At the third month recall, the swelling and pain had subsided completely (Fig. 2). The panoramic view also showed absence of radiolucency (Fig. 3).

## CASE 2

**Figs 4A to D:**
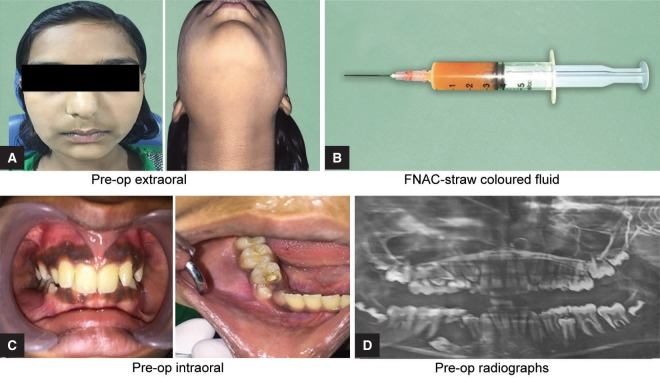
Preoperative photographs: (A) Extraoral photograph; (B) Fine needle aspiration colored fluid; (C) Intraoral photograph; (D) OPG

**Figs 5A to C:**
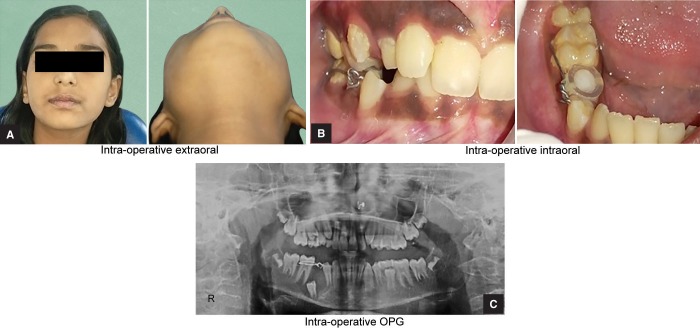
Intraoperative photographs: (A) Extraoral photograph; (B) Intraoral photograph; (C) OPG

**Figs 6A to C:**
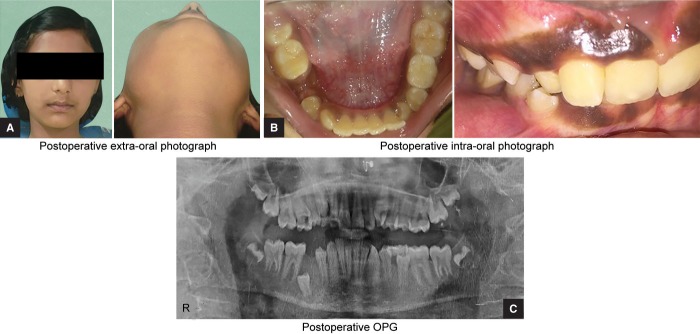
Postoperative photographs: (A) Extraoral photograph; (B) Intraoral photograph; (C) OPG

A 11-year-old-female patient reported to the Outward Patient Department of Pediatric and Preventive Dentistry (Pune, Maharashtra), with the chief complaint of pain and swelling in the lower right back region of the jaw since 20 days. Medical history was non-contributory. Extraorally facial asymmetry was noted on the lower right side of the jaw, extending antero-posteriorly from the angle of the mouth to the lower border of the mandible. Palpatory findings revealed the swelling was woody hard and tender on palpation. Intraorally expansion of the buccal cortical plate was seen extending from the region mesial to tooth 44 to the region distal to tooth 46. The overlying mucosa was hard in consistency and tender on palpation. Also, the involved primary tooth, i.e., 85, was grossly carious. There were no abnormalities associated with the soft tissues (Fig. 4). OPG examination revealed single, regular, unilocular, well-defined, corticated radiolucency involving erupting 44, laterally completely involving the impacted 45 further extending to the distal side of 47 measuring around 2.5 × 2.5 cm (Fig. 4). The treatment procedure carried out was the same as that of case report 1. The first recall was carried out within one week, and a reduction in tenderness and extraoral swelling was observed. Thereafter, the recall was carried out at one-month interval. A gradual reduction in the size of extraoral swelling and intraoral buccal cortical plate expansion was noticed within one month (Fig. 5). Clinical as well as panoramic radiographic examinations were performed every time when the patient was recalled. At the third-month recall visit, the intraoral swelling appeared to have subsided (Fig. 6).

## DISCUSSION

Dentigerous cyst is also known as “follicular cyst”. An odontogenic cyst of developmental origin. Most of the times it was an accidental finding when radiographs are taken to investigate the adjacent tooth that was either infected, carious, or mal-aligned. The dentigerous cyst seems to enclose impacted, embedded, or submerged tooth by the expansion of its follicle. The normal follicular space is approximately 3–4 mm, whereas a dentigerous cyst can be suspected when the space is more than 5 mm.^2^ Dentigerous cyst on radiographic examination shows unilocular, radiolucent lesion characterized by well-defined sclerotic margins associated with the crown of an unerupted tooth. The dentigerous cyst can be classified into two types based on its origin, i.e., originated from reduced enamel epithelium (REE), they occur in 2nd and 3rd decades, and predominantly involves mandibular 3rd molars. Based on the presence or absence of inflammation into inflammatory and non-inflammatory, diagnosed in 1st and early 2nd decade (i.e., mixed dentition period), with mandible is more commonly involved than maxilla in the ratio of 10:1.^7^

Dental caries are considered as one of the most common etiological factors for periapical inflammation involving primary molars. There are many schools of thoughts for the development of inflammatory dentigerous cyst like, (1) accumulation of fluid (i.e., inflammatory exudate) between the reduced enamel epithelium and the enamel organ due to the spread of infection to the dental follicle leading to the development of a dentigerous cyst. (2) It is seen that seldom the crown of a permanent tooth might erupt into a radicular cyst of a primary precursor leading to the development of a dentigerous cyst. Shear in 1994 stated that most jaw cysts occur in the first decade of life.^8^ The prevalence of dentigerous cyst development within the jaw close to the region of the second primary molars has been connected to the observations that the second primary molars are associated with greater caries susceptibility. The close relationship between a second primary mandibular molar and the follicle of the successor has been associated with a facilitated spread of infection in comparison with other primary teeth.^8^

The cystic potential of radicular lesions particularly in primary dentition as a result of native irritation could induce adverse effects on the successor tooth follicle (da Silva et al.). These conditions are very common in primary molars. Thus, a close follow up protocol should be implemented bearing in mind that the pathological changes associated with an endodontically treated deciduous tooth may remain symptom-free. The materials used for dental treatment in deciduous teeth will trigger the expansion of cysts.^8^

Pathophysiology for inflammatory dentigerous cysts is obstruction of venous outflow, due to the pressure exerted by a potentially erupting tooth on the impacted follicle, which induces rapid transudation of serum through capillary walls. The increased hydrostatic pressure of this pooling fluid separates the follicle from the crown with or without reduced enamel epithelium.^9^ The differential diagnosis of the dentigerous cyst includes ameloblastoma, odontogenic keratocyst, odontogenic fibroma, odontogenic myxoma, cementomas, and Pindborg tumor. Early identification and removal is necessary as they may rarely have the potential to develop into odontogenic tumors like ameloblastoma and malignancy like squamous cell carcinoma and mucoepidermoid carcinoma.^9,10^

The radiographic view of inflammatory dentigerous cyst shows spherical or ovoid, well-demarcated unilocular radiolucency at the border of the lower jaw.^8^ A cyst is sometimes related to the roots of a non-vital tooth and therefore the crown of an unerupted permanent successor with a sclerotic border. The histopathological image of cyst evaluated in these case reports showed inflammation with reduced enamel epithelial tissues. Hence, this case was diagnosed as inflammatory dentigerous cyst.^8^ According to the location of radiolucency around the crown of an unerupted tooth, there are three main varieties of dentigerous cyst and they are central, lateral, and circumferential types.^11^ Enucleation is the preferred treatment modality when there is no likelihood of damaging adjacent anatomical structures. On the other side, marsupialization will maintain disorder in its cavity, promote eruption of a tooth and conjointly minimize the danger of harm to special anatomical structures. Especially for young patients, the treatment modality should be as conservative as possible in order to decrease possible problems to the adjacent developing structures.^10^

The conservative treatment modality is extremely important in cases of huge lesions and once the permanent teeth concerned have eruptive potential. The marsupialization or Partsch's technique involves removing a window from the lesion and suturing the encircling mucoperiosteum to the margins of the cyst wall. The ensuing cavity is filled with gauze, which is removed after seven to ten days.^10^

Thomas modified Partsch's method; in his modification, a small opening is made in the defect and a soft metal or polyethylene tube for drainage is inserted and fixed at the site by attaching it to an adjacent tooth.^10^ This modality was called as the decompression technique. The procedure aims to reduce the size of the cyst. Opening the cyst lumen eliminates its osmotic pressure and bone apposition gradually occurs at the site, which was previously occupied by the epithelial covering of the cyst. The insertion of a device in cystic lumen is done in order to maintain patent communication between the interior of the cyst and the oral cavity. This procedure characterizes the decompression and differs from marsupialization because this technique involves the biopsy of large fragments of tissues.^12,13^

## CONCLUSION

Periapical radiolucency associated with primary teeth is often misdiagnosed either as an infected radicular cyst of a preceding tooth or a dentigerous cyst of the successor. It is very common for a dentigerous cyst to mimic an infected radicular cyst, especially when associated with a carious primary tooth as well as its unerupted permanent successor. Also, a carious primary tooth may have the potential to involve in the development of inflammatory dentigerous cysts and thus proper diagnosis is important to plan a proper treatment protocol. Decompression is the most conservative technique which results in significant reduction of the cyst size.^13^ Thus, to conclude, the decompression technique can be considered as a conservative treatment of choice since the preservation of the permanent successor was desirable.

## CLINICAL SIGNIFICANCE

This technique can be employed in situations when the underlying permanent tooth needs to be retained. Some other advantages of this technique are minimal bone loss with faster wound healing. Thus, to summarize, this technique is the most conservative with minimal limitations compared to other techniques.
